# Supplementary material for: Low thermal contact resistance boron nitride nanosheets composites enabled by interfacial arc-like phonon bridge
Source: Nat Commun. 2024 Apr 4;15:2905. doi: 10.1038/s41467-024-47147-1 (PMC10994942; doi:10.1038/s41467-024-47147-1)
Supplement: Supplementary file 1 — Supplementary Information [file 41467_2024_47147_MOESM1_ESM.docx]

**Low thermal contact resistance boron nitride nanosheets composites enabled by interfacial arc-like phonon bridge**

Ke Zhan^1,11^, Yucong Chen^1,11^, Zhiyuan Xiong^2,12^, Yulun Zhang^1^, Siyuan Ding^1^, Fangzheng Zhen^3^, Zhenshi Liu^4^, Qiang Wei^5^, Minsu Liu^1,3,6^, Bo Sun^1,7^, Hui-Ming Cheng^8,9,10,12^ and Ling Qiu^1,7,12^

^1^Shenzhen Geim Graphene Center (SGC), Tsinghua-Berkeley Shenzhen Institute (TBSI) & Tsinghua Shenzhen International Graduate School (TSIGS), Tsinghua University, Shenzhen 518055, China

^2^School of Light Industry and Engineering, South China University of Technology, Guangzhou 510614, China

^3^Monash Suzhou Research Institute (MSRI), Monash University, Suzhou 215000, China

^4^Sunwoda Electronic Co., Ltd., Shenzhen, 518108, China

^5^Vivo Mobile Communication Co., Ltd., Dongguan 523860, China

^6^Foshan (Southern China) Institute for New Materials, Foshan, 528200, China

^7^Institute of Materials Research, Tsinghua Shenzhen International Graduate School, Guangdong Provincial Key Laboratory of Thermal Management Engineering and Materials, Shenzhen, Guangdong 518055, China

^8^Shenzhen Key Lab of Energy Materials for Carbon Neutrality, Shenzhen Institute of Advanced Technology, Chinese Academy of Sciences,1068 Xueyuan Road, Shenzhen, 518055, China.

^9^Faculty of Materials Science and Energy Engineering, Shenzhen University of Advanced Technology, 291 Louming Road, Shenzhen, 518107, China.

^10^Shenyang National Laboratory for Materials Science, Institute of Metal Research, Chinese Academy of Sciences, 72 Wenhua Road, Shenyang 110016, China.

^11^These authors contributed equally: Ke Zhan, Yucong Chen.

12These authors jointly supervised this work: Ling Qiu, Zhiyuan Xiong, Hui-Ming Cheng. e-mail: xzyscut@scut.edu.cn; cheng@imr.ac.cn; ling.qiu@sz.tsinghua.edu.cn

**This PDF file includes:**

Supplementary Text

Figs. S1 to S12

Tables S1 to S5

**Other Supplementary Materials for this manuscript include the following:**

Movie S1 and S2

**Supplemental Text**

**Ductile to brittle transition, and flexibility in BNNS-TIMs**

The cross point of curves of $G^{’}$(storage modulus) and $G^{’’}$(loss modulus) for BNNS-TIMs delays from strain at 10^-3^% to 10^-2^% as filler loading shifts from 90 wt.% to 50 wt.%. This indicates increased plasticity at lower filler loading, as the material is able to undergo larger strains before exhibiting significant energy dissipation. In addition, the compressive modulus, as observed from the compressive stress-strain curves, decreases continuously as the filler loading decreases. This suggests that the material becomes less stiff and more compliant with lower filler loading, further supporting the transition from a brittle to a more ductile property of the BNNS-TIMs as the filler loading decreases from 90 wt.% to 50 wt.%. Flexibility of BNNS-TIMs could be benchmarked by the figure of merit, as the equation shows, $f_{FoM}= \sigma_{y}/E$, where $\sigma_{y}$ is the yield strength and $E$ is the elastic modulus of the material^1^. BNNS-TIMs belongs to intermediate region between elastomer and polymer as shown in Figure S5, and the repetitious bending test for 1000 times at a bending radius of 2.5 mm cause negligible deterioration in through-film thermal conductivity of BNNS-TIMs at 70 wt.% loading.

**Figure S1.** Additional details for microstructure characterization of BNNS-TIMs

(A) SEM image showing parallel BNNSs on the cutting surface of BNNS-TIMs.

(B-C) Side-view of BNNS-TIMs with loadings of (B) 60 wt.% and (C) 80 wt.%, complementing Figures 1D-F.

(D) Average orientation angle of fillers derived from the XRD patterns for BNNS-TIMs at loadings of 50-90 wt.%. The scale bar for (A-C) is 20 μm.

(E) Fourier Transform Infrared Spectroscopy (FTIR) spectra on polymer matrix, BNNS powder, and BNNS-TIM.

(F) Raman Spectra of BNNS-TIMs and raw h-BN powder as reference. Raman spectra captured when the laser beam targets the cutting face of BNNS-TIMs, where the BNNS are aligned parallel (C1// & C2//), and the internal region, where the BNNS are oriented perpendicular (C1⊥ & C2⊥).

(G) Structural formula of polymer matrix which is an acrylic acid-butyl acrylate-2-ethylhexyl acrylate copolymer, of which the chemical formula is (C_11_H_20_O_2_·C_7_H_12_O_2_·C_3_H_4_O_2_)_x_.

**Figure S2.** Additional mechanical properties for BNNS-TIMs and polymer matrix

(A) Rheology property of BNNS-TIMs at 50/90 wt.% loadings and pure polymer matrix, showing $G^{’}$(storage modulus) and $G^{’’}$(loss modulus) at different strains.

(B) Stress-strain curves describing the compressive behavior of BNNS-TIMs at 50-90 wt.% loadings and pure polymer matrix.


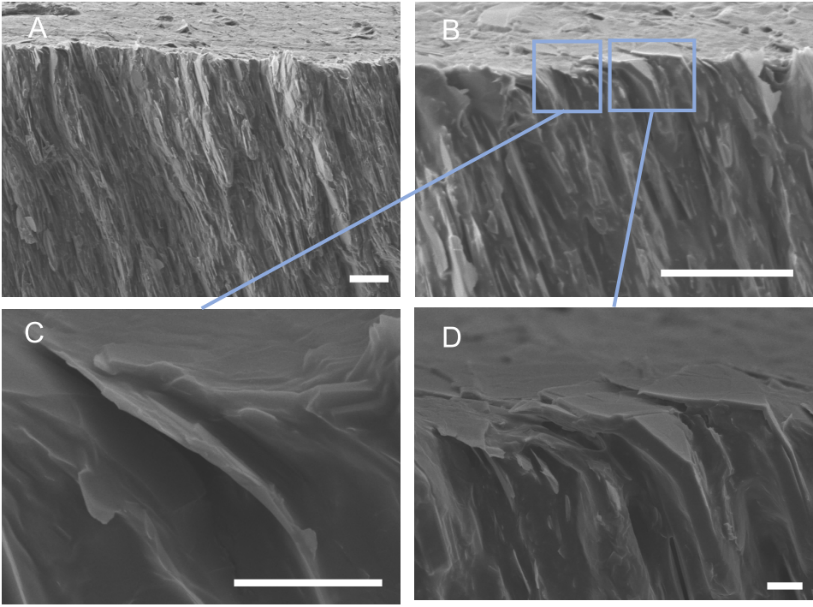


**Figure S3.** SEM images showing the structure near the cutting surface of 60 wt.% specimens that were processed under immersion in liquid nitrogen (-196^o^C)

(A-B) The rotation of nanosheets seen for room temperature processing is absent.

(C-D) Only bending of nanosheets is observed, when the processing temperature is below the glass-transition temperature, due to the increased brittleness of the polymer matrix. The scale bar for (A-B) is 20 μm, and for (C-D) is 2 μm.


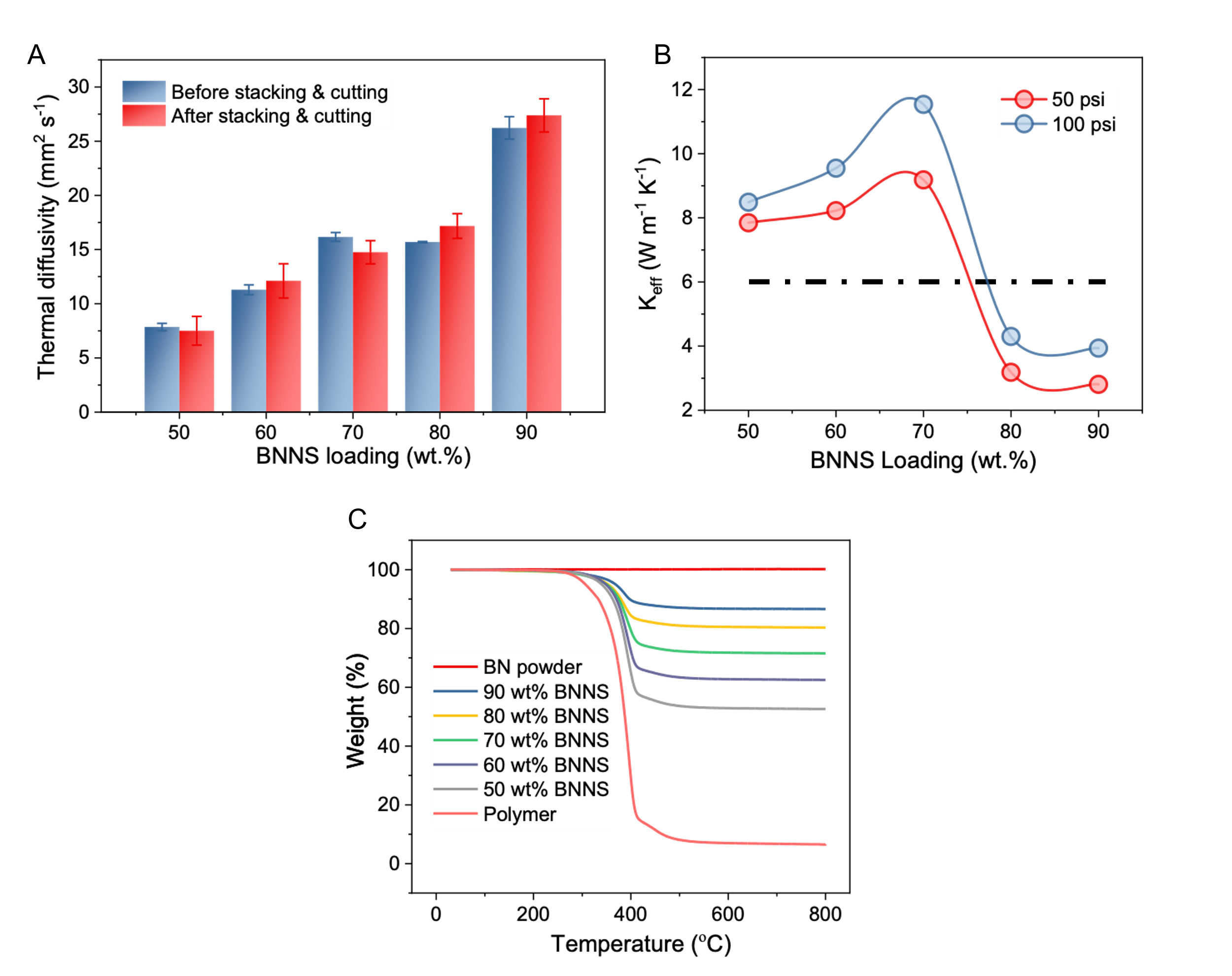


**Figure S4.** Additional thermal characterization on BNNS-TIMs

(A) Thermal diffusivity of BNNS/polymer composites films and BNNS-TIMs at loadings of 50 to 90 wt.%.

(B) $K_{eff}$ of BNNS-TIMs at loadings of 50 to 90 wt.%.

(C) Results of the thermogravimetric analysis of BNNS-TIMs at loadings of 50 to 90 wt.%, pristine h-BN powder and the pure polymer matrix.

**Figure S5.** Demonstration of the bendability of BNNS-TIMs at 70 wt.% loading with a thickness of 0.5mm following the widely-adopted method^2,3^

(A) Thermal diffusivity of BNNS-TIMs at different bending angles with corresponding bending radius.

(B) Thermal diffusivity of BNNS-TIMs after bending for 1/10/100/1000 times at bending angle of 170^o^.

(C) Benchmark of BNNS-TIM in demonstrating the flexibility^1^.

**
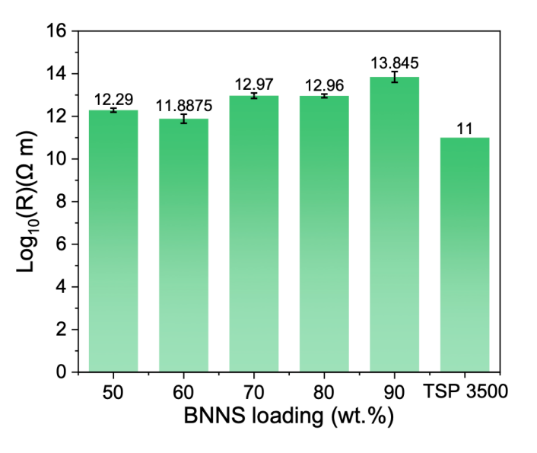
**

**Figure S6.** Volume resistivity of BNNS-TIMs at loadings of 50 to 90 wt.%, and commercially available dielectric TIMs (Henkel TSP 3500).

**Figure S7.** SEM images of side-views of BNNS-TIMs at 60 wt.% loading with thicknesses of (A) 200 μm and (B) 50 μm, indicating the arc-like structure is not thickness-dependent. (C) Surface roughness (Sa, Arithmetic Mean Deviation) of BNNS-TIMs across various filler loadings ranging from 50 to 90 wt.%. Inset at the top-right corner is a 3D reconstructed image of a 70 wt.% BNNS-TIM sample surface.

**Figure S8.** Additional details to support non-equilibrium molecular dynamic (NEMD) simulation

(A) The simulation box at equilibrium for contact angles of (A_1_) 30°, (A_2_) 45°, (A_3_) 60° and (A_4_) 90°.

(B) Thermal conductance at the interfaces adjacent to the hot (red bar) and cold sides (cold bar) for contact angles of 15°, 30°, 45°, 60° and 90°.

(C) The interatomic bonding at the interface of BNNSs contact at angles of 30°, 45°, 60 °and 90°, compared to the covalent bonding of BNNS in the basal plane.

(D) The simulation box at equilibrium for the contact angles of 15°, demonstrating that the actual contact angle between parallel and tilted BNNS is greater than the preset angle of 15°.





**Figure S9.** SEM images on BNNS-TIMs with a filler loading of 60 wt.% obtained from continuous manufacturing

(A) Side-view images show apparent nanosheet rotation.

(B) Cut surface shows nanosheets parallel to the film. The scale bars are 50 μm.


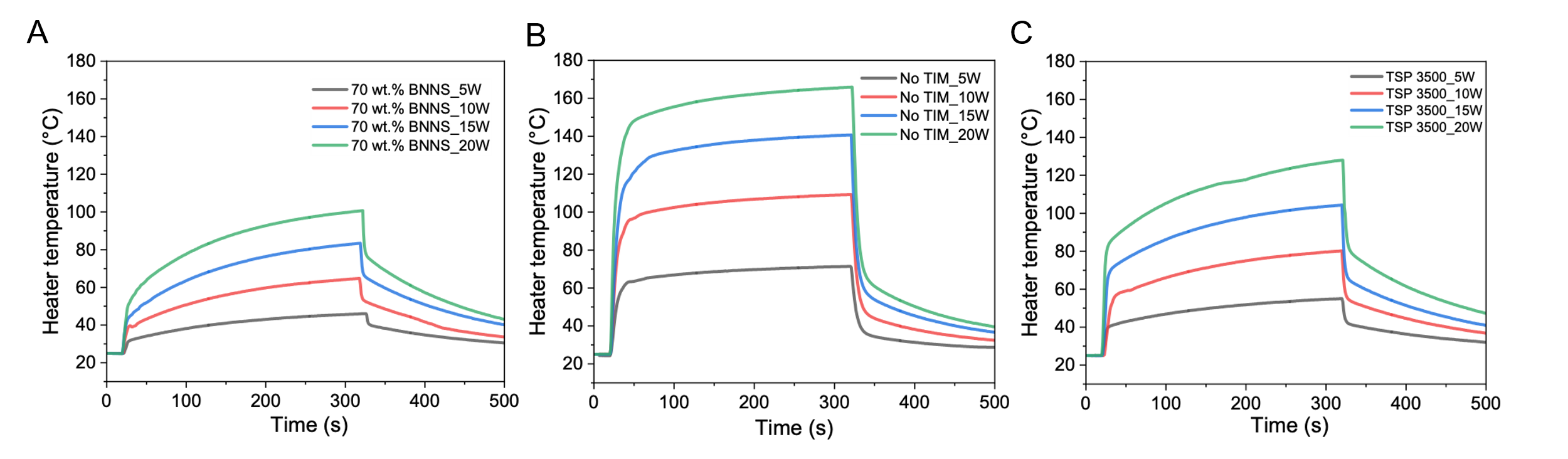


**Figure S10.** Primitive temperature-time curves for Figure 5C that demonstrate the performance of BNNS-TIMs on electronics cooling. (A) 70 wt.% BNNS. (B) No TIM. (C) Commercial TSP 3500.

**
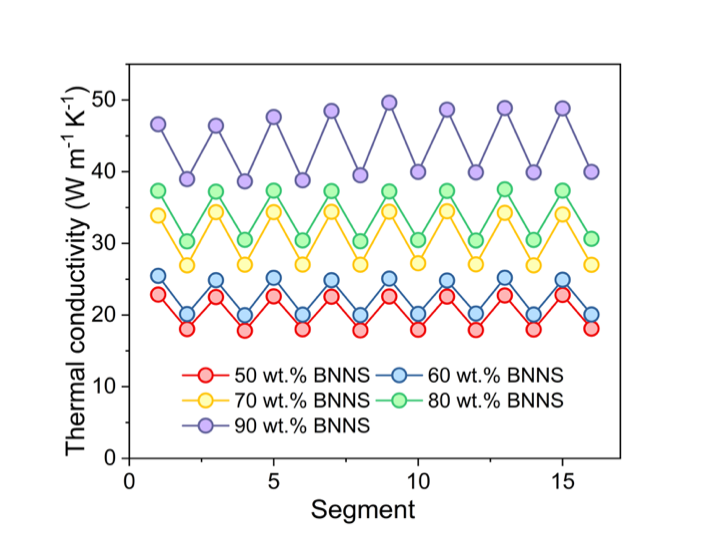
**

**Figure S11.** Thermal conductivity of BNNS-TIMs at loadings of 50-90 wt*.*% with temperature shock between 25^o^C and 100^o^C for eight cycles.

**Figure S12**. FEM simulation results based on isotropic elasticity that show (A) instant stress and (B) cumulative strain distribution in specimens with 50, 70, and 90 wt.% loadings under an external shear force. These results show a similar trend to Figure 2C-D, in cumulative strain pattern as filler loading increase, though with minor variations in strain, further explaining the nanosheet rotation behavior under shear cutting.

**Table S1.** Summary of the calculation of the average orientation angle α of the BNNSs in the composites.

| (hkl) | Crystalline plane angle $\boldsymbol{\alpha}_{\boldsymbol{hkl}}$ |  | BNNS loading | $\boldsymbol{\alpha}_{\boldsymbol{1}}$ | $\boldsymbol{\alpha}_{\boldsymbol{2}}$ | $\boldsymbol{\alpha}_{\boldsymbol{3}}$ | $\boldsymbol{\alpha}_{\boldsymbol{4}}$ | $\boldsymbol{\alpha}_{\boldsymbol{5}}$ | $\bar{\boldsymbol{\alpha}}$ |
| --- | --- | --- | --- | --- | --- | --- | --- | --- | --- |
| 002 | 0° |  | 50 wt.% | 53.34° | 74.10° | 66.88° | 56.92° | 53.96° | 61.04° |
| 100 | 90° |  | 60 wt.% | 84.42° | 87.97° | 88.34° | / | / | 86.91° |
| 101 | 74.21° |  | 70 wt.% | 80.80° | 83.52° | 87.97° | / | / | 84.10° |
| 102 | 60.52° |  | 80 wt.% | 63.31° | 82.04° | 86.03° | / | / | 77.13° |
| 004 | 0° |  | 90 wt.% | 78.83° | 87.33° | 88.13° | 83.10° | / | 84.35° |

**Table S2.** Physical parameters required for the calculation of thermal conductivity from the thermal diffusivity of BNNS-based TIMs.

| BNNS loading  (wt.%) | Density  (g cm^-3^) | Heat capacity  (J g^-1^ K^-1^) | Thermal  diffusivity  (mm^2^ s^-1^) | Thermal conductivity  (W m^-1^ K^-1^) |
| --- | --- | --- | --- | --- |
| 50 | 1.378 | 1.367 | ~8 | ~12 |
| 60 | 1.509 | 1.27 | ~12 | ~22 |
| 70 | 1.62 | 1.227 | ~14 | ~28 |
| 80 | 1.78 | 1.083 | ~18 | ~32 |
| 90 | 1.86 | 0.999 | ~26 | ~50 |

**Table S3.** Summary of reported studies on h-BN based TIMs, specializing in the comparison of the materials, methods, filler loading, and through-film thermal conductivity.

| Filler/Matrix | Methods | Loading | Through-film thermal conductivity  (W m^-1^ K^-1^) | Year |
| --- | --- | --- | --- | --- |
| BNNS/Polyolefin | Roll milling | 43.75 vol.% | 6.94 | 2017^4^ |
| BN/PVDF | Cutting & stacking | 30 wt.% | 3.5 | 2019^5^ |
| BN/Rubber | Cutting & stacking | 60 wt.% | 7.62 | 2021^6^ |
| BN/Rubber | Cutting & stacking | 45 wt.% | 5.4 | 2018^7^ |
| BN/Epoxy | Vacuum-assisted | 44 vol.% | 9 | 2017^8^ |
| SiC/BNNS/Epoxy | Vacuum-assisted | 21.9 vol.% | 4.22 | 2020^9^ |
| TiO2@BN/PUA | Electric field | 20 vol.% | 1.54 | 2016^10^ |
| BN/Vinyl silicone oil | Shear field | 60 wt.% | 5.65 | 2022^11^ |
| BNNS/PVP | Magnetic field | 74 wt.% | 12.1 | 2022^12^ |
| FeCo@BNNS/PDMS | Magnetic field | 50 vol.% | 2.25 | 2019^13^ |
| BN/Epoxy | Salt-template | 73 wt.% | 6.1 | 2020^14^ |
| BNNS/Epoxy | Freeze drying | 32 vol.% | 4.38 | 2020^15^ |
| BNNS/PU | Freeze drying | 85 wt.% | 11.5 | 2022^16^ |
| BNNS/Epoxy | Bi-directional freezing | 15 wt.% | 3.87 | 2020^17^ |
| BN/LCP | 3D Printing | 20 wt.% | 1.77 | 2022^18^ |
| BNNS/PDMS | 3D Printing | 50 wt.% | 5.65 | 2019^19^ |
| BNNS/Polymer | Stacking-cutting | 90 wt.% | 50 | **This work** |

**Table S4.** Data for the radar chart in Figure 3D which comprehensively compares the thermal, electrical, and mechanical properties to highlight the advantages of this work over reported studies and commercially available products.

| Type of TIM | Thermal conductivity (W m^-1^ K^-1^) | Thermal resistance (in^2^ K W^-1^) | K_eff_  (W m^-1^ K^-1^) | Hardness | Volume resistivity (Ω m) | Dielectric strength (kV mm^-1^) |
| --- | --- | --- | --- | --- | --- | --- |
| This work | 25 | 0.18 | 9.4 | 62 A | 10^12 | 21 |
| BN network^20^ | 0.94 | 0.618 | 0.915 | / | / | / |
| Henkel TSP3500 | 3.5 | 0.33 | 3.5 | 90 A | 10^11 | 8 |
| Chomerics Gap filler pad 976 | 6.5 | 0.3 | 5.167 | 10A | 10^12 | 5.1 |
| Honeywell TIP3500 | 3.5 | 0.36 | 4.306 | 80 A | 10^11.7 | 10 |
| Graphite^21^ | 80 | 0.307 | 5.05 | / | / | / |
| CNT^22^ | 0.4 | 0.17 | 1.824 | / | / | / |

**Table S5.** Physical parameters and electric/thermal performance of BNNS-TIMs with a filler loading of 70 wt.% obtained by continuous manufacturing.

| Sample Thickness  (mm) | Density  (g cm^-3^) | Breakdown voltage (kV) | Dielectric strength (kV mm^-1^) | Total Thermal resistance  (K in^2^ W^-1^) |
| --- | --- | --- | --- | --- |
| 0.2 | 1.605 | 6.2 | 31 | 0.042 |
| 0.5 | 1.631 | 7.9 | 15.8 | 0.070 |
| 1.0 | 1.636 | 7.8 | 7.8 | 0.121 |

**References**

1. Peng, J. & Snyder, G. J. A figure of merit for flexibility. Science 366, 690–691 (2019).

2. Zheng, Z. et al. Pre‐Buried Additive for Cross‐Layer Modification in Flexible Perovskite Solar Cells with Efficiency Exceeding 22%. Adv Mater 34, 2109879 (2022).

3. Li, L. et al. Flexible all-perovskite tandem solar cells approaching 25% efficiency with molecule-bridged hole-selective contact. Nat Energy 7, 708–717 (2022).

4. Feng, C.-P. et al. Electrically insulating POE/BN elastomeric composites with high through-plane thermal conductivity fabricated by two-roll milling and hot compression. Adv Compos Hybrid Mater 1, 160–167 (2018).

5. Song, Q. et al. Enhanced through-plane thermal conductivity and high electrical insulation of flexible composite films with aligned boron nitride for thermal interface material. Compos Part Appl Sci Manuf 127, 105654 (2019).

6. Hu, Q. et al. Oriented BN/Silicone Rubber Composite Thermal Interface Materials with High Out-of-Plane Thermal Conductivity and Flexibility. Compos Part Appl Sci Manuf 152, 106681 (2021).

7. Xue, Y. et al. Improvement in thermal conductivity of through-plane aligned boron nitride/silicone rubber composites. Mater Design 165, 107580 (2019).

8. Yu, C. et al. Enhanced through-plane thermal conductivity of boron nitride/epoxy composites. Compos Part Appl Sci Manuf 98, 25–31 (2017).

9. Xiao, C. et al. Epoxy composite with significantly improved thermal conductivity by constructing a vertically aligned three-dimensional network of silicon carbide nanowires/ boron nitride nanosheets. Compos Part B Eng 187, 107855 (2020).

10. Kim, K., Ju, H. & Kim, J. Filler orientation of boron nitride composite via external electric field for thermal conductivity enhancement. Ceram Int 42, 8657–8663 (2016).

11. Niu, H. et al. Vertical Alignment of Anisotropic Fillers Assisted by Expansion Flow in Polymer Composites. Nano-micro Lett 14, 153 (2022).

12. He, H. et al. Microstructured BN Composites with Internally Designed High Thermal Conductivity Paths for 3D Electronic Packaging. Adv Mater 34, 2205120 (2022).

13. Yuan, J., Qian, X., Meng, Z., Yang, B. & Liu, Z.-Q. Highly Thermally Conducting Polymer-Based Films with Magnetic Field-Assisted Vertically Aligned Hexagonal Boron Nitride for Flexible Electronic Encapsulation. Acs Appl Mater Inter 11, 17915–17924 (2019).

14. Xu, X. et al. 3D boron nitride foam filled epoxy composites with significantly enhanced thermal conductivity by a facial and scalable approach. Chem Eng J 397, 125447 (2020).

15. Ghosh, B. et al. Highly Ordered BN⊥–BN⊥ Stacking Structure for Improved Thermally Conductive Polymer Composites. Adv Electron Mater 6, 2000627 (2020).

16. Zhao, N., Li, J., Wang, W., Gao, W. & Bai, H. Isotropically Ultrahigh Thermal Conductive Polymer Composites by Assembling Anisotropic Boron Nitride Nanosheets into a Biaxially Oriented Network. Acs Nano 16, 18959–18967 (2022).

17. Huang, T., Li, Y., Chen, M. & Wu, L. Bi-directional high thermal conductive epoxy composites with radially aligned boron nitride nanosheets lamellae. Compos Sci Technol 198, 108322 (2020).

18. Luo, F. et al. Orientation behavior and thermal conductivity of liquid crystal polymer composites based on Three-Dimensional printing. Compos Part Appl Sci Manuf 160, 107059 (2022).

19. Liang, Z. et al. General, Vertical, Three-Dimensional Printing of Two-Dimensional Materials with Multiscale Alignment. Acs Nano 13, 12653–12661 (2019).

20. Zhou, S. et al. Expansion force induced in situ formation of a 3D boron nitride network for light-weight, low- k , low-loss, and thermally conductive composites. J Mater Chem A 10, 14336–14344 (2022).

21. Li, J. et al. Thermal Interface Materials with Both High Through-Plane Thermal Conductivity and Excellent Elastic Compliance. Chem Mater 33, 8926–8937 (2021).

22. Ping, L. et al. Clean, fast and scalable transfer of ultrathin/patterned vertically-aligned carbon nanotube arrays. Carbon 133, 275–282 (2018).
