# Supplementary material for: Low thermal contact resistance boron nitride nanosheets composites enabled by interfacial arc-like phonon bridge
Source: Nat Commun. 2024 Apr 4;15:2905. doi: 10.1038/s41467-024-47147-1 (PMC10994942; doi:10.1038/s41467-024-47147-1)
Supplement: Supplementary file 3 — Description of Additional Supplementary Files [file 41467_2024_47147_MOESM3_ESM.pdf]

Description of Additional Supplementary Files

Supplementary Movie 1: Flexibility of 70wt.% BNNS-TIM

Supplementary Movie 2: Continuous manufacturing of 70 wt.% BNNS-TIMs
