# Supplementary material for: Low thermal contact resistance boron nitride nanosheets composites enabled by interfacial arc-like phonon bridge
Source: Nat Commun. 2024 Apr 4;15:2905. doi: 10.1038/s41467-024-47147-1 (PMC10994942; doi:10.1038/s41467-024-47147-1)
Supplement: Supplementary file 6 — Reporting Summary [file 41467_2024_47147_MOESM6_ESM.pdf]

nature research

Corresponding author(s): Ling Qiu

Lasing Reporting Summary

Nature Research wishes to improve the reproducibility of the work that we publish. This form is intended for publication with all accepted papers reporting claims of lasing and provides structure for consistency and transparency in reporting. Some list items might not apply to an individual manuscript, but all fields must be completed for clarity.

For further information on Nature Research policies, including our data availability policy, see Authors & Referees.

Experimental design

Please check: are the following details reported in the manuscript?

1. Threshold

Plots of device output power versus pump power over a wide range of values indicating a clear threshold

☐ Yes
☒ No

This work uses standard Raman Spectrometer and Laser Flash Apparatus for materials characterization, the "Threshold" information is not relevant.

2. Linewidth narrowing

Plots of spectral power density for the emission at pump powers below, around, and above the lasing threshold, indicating a clear linewidth narrowing at threshold

☐ Yes
☒ No

This work uses standard Raman Spectrometer and Laser Flash Apparatus for materials characterization, the "Linewidth narrowing" information is not relevant.

Resolution of the spectrometer used to make spectral measurements

☒ Yes
☐ No

The model number of Raman Spectrometer is given as "Raman spectra were collected on a high-resolution analytical Raman microscope (Horiba LabRAM HR80, Japan) using 532 nm laser excitation." in the page 15 of the main text, in which the resolution of the spectrometer could be tracked by model number.

3. Coherent emission

Measurements of the coherence and/or polarization of the emission

☐ Yes
☒ No

This work uses standard Raman Spectrometer and Laser Flash Apparatus for materials characterization, the "Coherent emission" information is not relevant.

4. Beam spatial profile

Image and/or measurement of the spatial shape and profile of the emission, showing a well-defined beam above threshold

☐ Yes
☒ No

This work uses standard Raman Spectrometer and Laser Flash Apparatus for materials characterization, the "Beam spatial profile" information is not relevant.

5. Operating conditions

Description of the laser and pumping conditions
Continuous-wave, pulsed, temperature of operation

☒ Yes
☐ No

For Laser Flash Apparatus, the temperature of operation is stated as "A laser flash apparatus (LFA 467, Netzsch, Germany) was used to study the thermal properties of the thermal conductive pads at 25C.", but for the other "Operating conditions" information is not relevant.

Threshold values provided as density values (e.g. W cm^{-2} or J cm^{-2}) taking into account the area of the device

☐ Yes
☒ No

This work uses standard Raman Spectrometer and Laser Flash Apparatus for materials characterization, the "Threshold values" information is not relevant.

6. Alternative explanations

Reasoning as to why alternative explanations have been ruled out as responsible for the emission characteristics
e.g. amplified spontaneous, directional scattering; modification of fluorescence spectrum by the cavity

☐ Yes
☒ No

This work uses standard Raman Spectrometer and Laser Flash Apparatus for materials characterization, the "Alternative explanations" information is not relevant.

7. Theoretical analysis

Theoretical analysis that ensures that the experimental values measured are realistic and reasonable
e.g. laser threshold, linewidth, cavity gain-loss, efficiency

☐ Yes
☒ No

This work uses standard Raman Spectrometer and Laser Flash Apparatus for materials characterization, the "Theoretical analysis" information is unnecessary to provide in the manuscript.

8. Statistics

Number of devices fabricated and tested

☒ Yes
☐ No

For Raman Spectrometer test, the number of materials being fabricated and tested is shown in Figure S1F in the Supporting Information (Page 3), with corresponding figure caption. And for Laser Flush Apparatus, the information is shown in Figure 4A in the main text (Page 9), with corresponding figure caption.

March 2018

Statistical analysis of the device performance and lifetime (time to failure)	☐ Yes<br>☒ No	This work uses standard Raman Spectrometer and Laser Flash Apparatus for materials characterization, the "device performance and lifetime" information is not relevant.
